# Medical News and Bulletin

**Published:** 1843-05-13

**Authors:** 


In the number of the Western and Southern Medical Recorder, for March, 1843, edited by Professor Cross, an omission has occurred in not attributing the Bibliographical Notice of Dr. Kirkbride's Report to this Journal. The identical notice there published will be found in the fourth number of the Medical Examiner for the current year. We have become inured to the constant appropriation of our Foreign Summary by contemporaries, often prepared at the cost of much time and labour; but our toleration does not yet extend to the adoption, without credit, of our own thoughts and words.

---

We have received an Introductory Lecture delivered at the Castleton Medical College, on the 10th April, 1843, by JAMES McCLINTOCK, M. D., President of the College, and Professor of Anatomy and Surgery, &c. After giving a highly flattering account of the Institution, the Doctor proceeds to direct the attention of the students to the importance of the study of anatomy, and enforces his views with spirit and good taste.

---

#### STATE OF THE OVARIES DURING MENSTRUATION.

Some recent writers have alleged that each act of menstruation is accompanied with the rupture of one of the Graafian vesicles. In a case of very rapidly fatal fever, occurring in a young woman immediately after one of the catamenial periods,—reported by M. Devay in the L'Experience, No. 296—the following appearances were found on dissection. The outer surface of the right ovary was injected, and presented towards its upper edge a small lacerated opening, in which there lay a minute coagulum of blood that was still soft. On cutting through the substance of the ovary, there was observed another cicatricula of somewhat less recent date, and which contained a small sanguineous concretion of the size of a hempseed: three Graafian vesicles were counted.

The left ovary exhibited several scars of older date. The right Fallopian tube contained a little fluid blood; the left one was quite empty. Some sanguinolent mucus was observed on the inner surface of the uterus.—*Medico-Chirurgical Review.* April, 1843.

---

#### MUTUAL FRIENDSHIP OF DISTINGUISHED ANATOMISTS AND PAINTERS.

It is well known that the great painters in the age of Leo X. devoted much of their attention to the study of anatomy. Leonardi da Vinci made the drawings for the Anthropotomia of Della Torre, and Michael Angelo those for the similar work of his friend Colombus. The prince of painters, the divine Raffaelle di Urbino, devoted much of his time to the composition of a work on human and comparative myology; and on the other hand, Francesco Fresconi, the physician of three successive Popes, was a most accomplished sculptor; and Berenger de Carpi, one of the earliest advocates of the use of mercurial fumigations in the treatment of syphilis, was so ardent an admirer of the fine arts that, on several occasions, he would accept of no other fee except a fine painting or statue from his patients. Vasari tells us that the Cardinal Colonna was obliged to give a celebrated painting by Raphael to the art-loving doctor, in payment for his professional aid.

Titian, with the assistance of one of his pupils, sketched the admirable drawings for the great work on anatomy by Vesalius. The great painter of Venice fully appreciated the merits of the young anatomist; for we find that, when he had finished the portraits of Charles V., Francis I., the Grand Signor Soliman and other crowned heads, he immediately commenced those of Vesalius and Ariosto; the aristocracy of talent appearing to him worthy of a place beside the aristocracy of rank.—*Ibid.*

---

#### M. ROUX'S HOSPITAL PRACTICE: LITHOTOMY AND LITHOTRITY.

During the five years from 1836 to 1841, 24 patients have been operated upon for urinary calculus in the Hôtel Dieu; in six cases by lithotrity, and in 18 by lithotomy. In all the first set, the patients recovered; but of the 18 who were cut, seven died.

In 1841, seven calculous patients were admitted under the care of M. Roux: in one case, that of a young girl eleven years of age, death took place on the very day of her admission, in consequence of a profuse diarrhœa, which had utterly wasted her strength. Of the other six patients, four were treated by lithotomy and two by lithotrity: of the entire number only one—one of the four—recovered. In one unfortunate case of lithotrity, the *brise-pierre* instrument had formed a false passage about the neck of the bladder. (Frightful mortality on the whole.) *Ibid.*

---

#### THE GOOD OF EMETICS.

The great despot of France, Louis XIV., lay ill in 1658 of a fever, for which, as a last resource, his medical attendants ordered antimonial wine. The king inquired if it was the advice of his minister that he should take it; and, on being answered in the affirmative, he swallowed the draught with a confidence that tended to insure its good effect. He grew better from that time, and in six days was pronounced convalescent. The minister of the day was Mazarin, who, in the following year, concluded the marriage between his sovereign and the Infanta of Spain, and gave to Europe the boon of repose by the peace of the Pyrenees. Not long afterwards Mazarin himself died from the effects of a remedy similar to that which had cured the king, which gave occasion for the rather puzzling remark that "an emetic had twice saved France!"

---

#### THE WAY TO MAKE A LONG STORY SHORT.

Boyer was one day consulted by a woman of loose character, who affected a great deal of modesty. Wearied out with her affectation in the recital of her complaints, he said bluntly, "Madam you have had the venereal disease!" "What do you mean, Sir?" said the dame indignantly; "never, I can assure you!" "Then you have it now," said the surgeon. "You are mistaken, Sir." "Well then you will have it!" and with this the conversation terminated.—*Lond. Lancet.*

